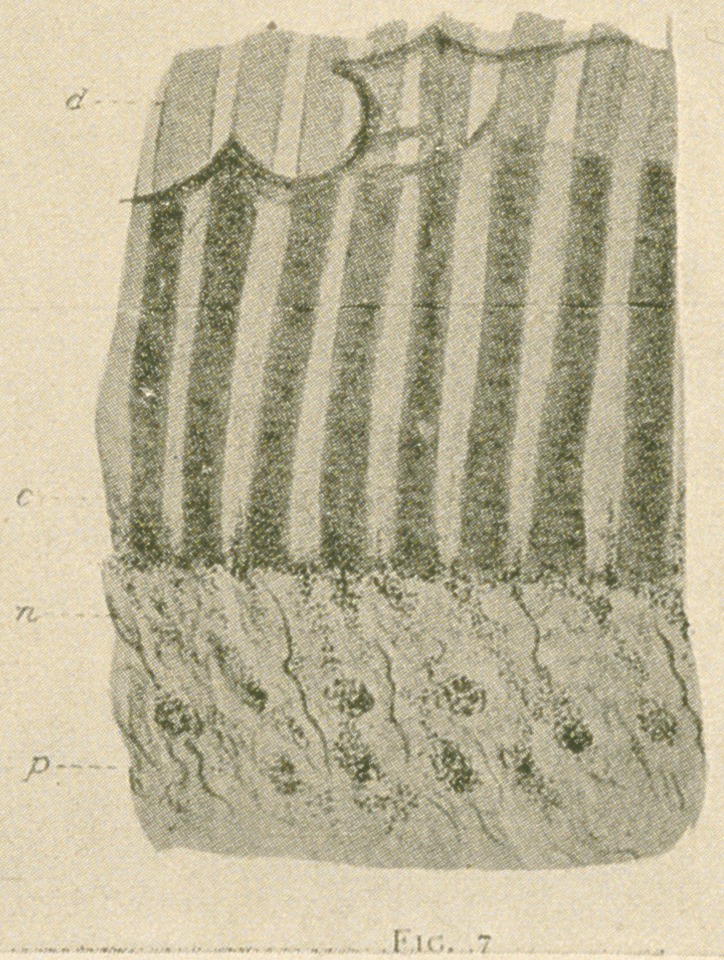# The Distribution of the Nerves of the Dental Pulp

**Published:** 1912-08-15

**Authors:** J. Howard Mummery


### THE DISTRIBUTION OF THE NERVES OF THE DENTAL PULP.

BY J. HOWARD MUMMERY, M.R.C.S., L.D.S.

Communicated by Prof. J. Symington, F. R. S., to the Royal Society, February 1, 1912.

The great improvements in the methods of histological research which have been developed during the last fifty years have very largely added to our knowledge of the peripheral distribution of the nerves in the various tissues and organs of the body.

The mode, however, in which sensory impressions conveyed from the hard dentine of the human tooth, which clinical experience shows to be highly endowed with sensibility, is not yet thoroughly understood. The difficulties attending the investigation of the relations between the nerve terminations and the calcified dental tissues are perhaps greater than those met with in tracing nerve tissue in other parts of the body, chiefly owing to the delicate connection between the soft pulp and the dentine, and the confusing optical effects produced in the latter tissue by its tubular structure.

Teeth have either to be prepared by hardening the pulp to such a degree that it can be ground down with the calcified tissue, as in the balsam process of Wiel, or they must be decalcified to enable the pulp and dentine to be cut together in the microtome. It is, no doubt, owing to these difficulties that the distribution of the ultimate nerve-fibrils of the pulp has so long been one of the problems of histology.

For the last fifty years very many attempts have been made to solve this problem, and many theories have been propounded, but none of these have been generally accepted, the evidence not having been considered sufficient by the majority of observers. It has, however, been satisfactorily shown that the bundles of medullated nerve-fibres which enter the tooth at the apical foramen lose their medullary sheath and spread out into a dense mass of fine fibres (fig. 1). This occurs at the periphery of the pulp and very abundantly at the coronal portion of the tooth, these non-medullated fibres forming an intricate plexus immediately beneath the odontoblast layer. This plexus, known as the plexus of Raschkow, can be well seen in suitably stained specimens, and consists entirely of non-medullated fibres (fig. 5).

From this plexus multitudes of fine neuro-fibrils course between and around the odontoblasts to the margin of the dentine, where many observers believe that they end, it having been suggested that they form a terminal plexus in this situation and that small, knob-like swellings can be detected, which some consider to be nerve end bodies.

This is the view held by Prof. Otto Fischer (1) in his lectures, published in 1909. He says: "That the pulp must be extraordinarily rich in nerve-fibres, daily practice convinces us. It is, however, still uncertain how far the terminal fibres extend, whether to the highly sensitive dentine enamel boundary or only as far as the odontoblast layer. From my own observations I must favor the latter view, which also is the predominating one at this time, for I have not in a single instance, by the use of the ordinary methods, seen nerve-fibres extending beyond the dentine cells."

Prof. Schafer (2) also says (1910): "The nerve-fibres are said to pass eventually between the odontoblasts and to end in arborisations close to the dentine, but they have not been followed into the dentinal tubules."

Magitot (3) described the nerve-fibres as entering the reticulate cells of the pulp which lie immediately beneath the odontoblasts and that these cells communicating with the odontoblasts by means of the pulp processes of the latter, a direct communication is established between the nerves and the odontoblasts. This statement of Magitot has not, however, been confirmed by any subsequent writer.

Another view is that the odontoblast cells really fulfill the function of nerve end organs. This is the view advocated by Mr. Hopewell-Smith (4), who, while allowing that the fact of the odontoblasts being of mesoblastic origin, while the nerves are developed from the epiblast, makes it impossible to consider them as ganglion cells, thinks they may still be considered to be sensation transmitters. No nerve-fibre has been seen to actually enter an odontoblast cell, although in teased-out preparations fine varicose fibres can be seen surrounding them (*loc. cit.*), but, in accordance with the neurone theory of Waldeyer, the interruption of anatomical continuity would not necessarily cause any interruption in the physiological path, and sensation could be transmitted without the actual continuity of cell and fibril.

Boll (5), as long ago as 1868, in studying this question, employed a $\frac{1}{3}$ per cent. solution of chromic acid. Examining fresh pulps treated in this manner, he found an immense number of fine fibres in communication with the nerve-fibres of the pulp, passing up to the dentine and projecting beyond the layer of odontoblasts; they appeared as if they had been pulled out from the tubes, but could not be shown to enter the dentine. His experiments were carried out on rodent teeth with persistent pulps.

Retzius (6), examining the teeth of young mice in 1894, says: "In vertical sections, the fibres, like a string of tiny beads, stretch between the odontoblast to the surface and there end free." He also says: "In tangential sections they can be partially traced into the dentine."

Carl Huber (7) traced nerve-fibres to the odontoblasts, and considered that they there terminated in free ends or granule-like bodies. He observed that these fibres surrounded the odontoblasts, enclosing them in a network, but that they made no connection with the cells, and he considered that they did not enter the dentine. His experiments were carried out on cats and rabbits, employing methylene blue injected into the carotid immediately after death.

Other researches are those of Dr. Michael Morgenstern (8), of Baden Baden, and Professor Romer (9) of Strasburg.

Morgenstern described nerves in the boundary between the enamel and dentine of permanent human incisor teeth in 1882, but could not then trace their connection with the nerve-fibres of the pulp. In his paper above referred to, published in 1892, he asserts that the dentine is supplied with nerves, not in all, but in many and very clearly-defined places. "The nerves," he says, "pass out of the pulp into the dentine, especially plentifully at the so-called horns of the pulp, as bundles of axis cylinders bound together by very little medullary substance." They course in nearly sheathless minute canals, in places of minute, in other places of greater calibre than the dentinal tubes, and cannot easily be distinguished from these by the ordinary methods of research. Each "nerve canal" contains two axis cylinders, from each of which a multitude of finer fibres pass out. The axis cylinders of one canal are, until they reach the neighborhood of the dentine-enamel and dentine-cementum boundaries, applied close to one another, they separate at that point by degrees from one another, divide and terminate in the dentine—under the junction of the enamel and cement, and in various ways in the enamel. Among other modes of termination, he considered that many fibres entered the spindle-like prolongations commonly seen at the dentine margin of the enamel, which he looked upon as nerve end bodies. He employed the Golgi method of staining. Oscar Romer (*loc. cit.*) came to the conclusion that "the nerves of the pulp penetrate as non-medullated fibres, the intervening spaces between the odontoblasts arrive in the zone between the odontoblasts and the dentine, and here penetrate into the anterior of the odontoblast, process, that is to say, into Kolliker's dentinal tubules. The chief mass of the nerve-filaments radiate out of the cupola of the pulp horns into the dentine, while the other zones of the dentine appear to be poorer in nerve-arms, and the dentine of the root seems to be entirely without nerves.

"A greater part of the dentinal tubes widen out at the enamel-dentine boundary into curious partly spindle-shaped partly club-shaped formations, which are chiefly arranged in very great numbers around the apices of the dentine cusps, and in which, in well preserved ground sections, small roundish or larger oval corpuscles are perceptible, which are often arranged in rosary-like rows and with gold chloride take an intense red stain. The small corpuscles in the interior of the knob-shaped enlargements of the dentinal tubules may be regarded, with great probability, as terminal corpuscles of sensitive nerves in the dentine and analogous to the terminal corpuscles of the sensory nerves of the skin and of the papillae of the mucous membranes."

These views, however, do not seem hitherto to have been accepted or corroborated, and the latest publications on the nerves of the pulp favor the view that the terminations are situated at the inner margin of the dentine.*

My own observations date from 1891, and have been made both on ground sections (Weil process) and on decalsified teeth. The method of Boll showed a great number of fibres reaching as far as the dentine margin; but the stain did not appear to give any definite nerve differentiation.

In Berlin in 1892, in company with the late Professor W. D. Miller, I tried the *intra vitam* staining with methylene blue—the teeth of a dog used in this experiment were broken up into as small fragments as possible, and examined under the microscope. In the adherent portions of pulp, an immense number of fine fibres were seen stained a deep blue,

*Since writing the above I have received the *Transactions* of the International Dental Congress at Berlin, in 1909, which has just been published, and in which a short paper appears by Professor Dependorf, of Leipzig. After describing the passage of nerve-fibres to the dentine and the plexus at its margin, he says he has traced nerve-fibres in many places into the odontogenetic zone, or area of partial calcification; he considers that this observation, however, cannot be taken as authoritative for the innervation of the dentine itself. No methods are given in the paper, and it is not accompanied by illustrations or photographs. ("A Contribution to the Knowledge of the Innervation of the Human Teeth, especially the Odontoblast Layer and the Dentine." "Verhandlungen des V. Internationalen Zahnarztlichen Kongresses," Berlin, 1909.)

and passing right up to the dentine, but could not be traced any further owing to the impossibility of obtaining sections of calcified dentine. The great abundance of these fine fibres, far greater in number than the dentinal fibrils, led to the conclusion that they were the fine nerve-fibres of the pulp, and that if sections could have been made they would have demonstrated the distribution of these fibres to the hard tissue.

I next made use of the iron and tannin impregnation method first described by Polaillon for nerve endings. The teeth made use of were chiefly bicuspid teeth from young subjects. They were decalcified after having been fixed in a solution of bichromate of ammonia, embedded in paraffin, and cut with the microtome.

The sections were placed in a 4 per cent. solution of per chloride of iron in water for ten minutes, washed in water and placed in a 2 to 4 per cent. solution of tannin in water and, when sufficiently blackened, dehydrated, cleared with clove oil, and mounted in balsam.

In these sections I was able to trace nerve-fibres from the medullated fibres of the pulp to the plexus beneath the odontoblasts, and from the plexus to the dentine-pulp boundary, where they could be seen passing between and around the odontoblast cells.

I examined these preparations carefully with Mr. Charles Tomes (10), and though it looked very much as if these fibres entered the dentine, we could neither of us feel certain that they did so. I did not resume my investigations until the beginning of the year 1911, when, in going through my old iron and tanning preparations, I found many of them apparently more perfectly impregnated than they had been in 1892, and several showed fine dotted fibres passing into the dentinal tubes.

I recorded this observation in a short note which I communicated to the Odontological Section of the Royal Society of Medicine (11). Although, however, I felt as the result of having seen these appearances several times that the true solution of the distribution of the nerve-fibres was here indicated, I was not in a position to bring forward any convincing evidence of this belief, or to show any specimens that I felt would carry conviction to others. From this date I undertook a renewed investigation of the subject with fresh tissue.

The iron and tannin process, while staining the nerve-fibres in a special manner, is not altogether satisfactory, as it stains the dentine so deeply that the course of the tubes cannot be very easily followed for any distance, and it also colors strongly other elements of the tissue than nerves.

Endeavoring to find a stain that would differentiate nerve-tissue more perfectly, I employed Benda's iron haematoxylin process. This method requires the preliminary use of a mordanting solution—the sections, hardened in formalin, are decalcified in nitric acid, treated with the mordant for twenty-four hours—and passed, after washing into a 1 per cent. solution of haematoxylin in water, where they are kept until they appear quite black. They are then transferred to a 10 per cent. solution of acetic acid in water and carefully watched until sufficiently differentiated.

Good sections treated by this method gave a very excellent demonstration of the nerves of the pulp, and showed very clearly the passage of fine nerve-fibres from the plexus of Raschkow around the odontoblasts to the dentine, where they could be seen as dotted lines entering the dentinal tubules in great numbers (fig 2.)

This method also shows very clearly the narrow marginal plexus at the dentine edge, the fibres being seen to pass laterally, parallel to the surface of the pulp, as well as forwards into the tubes. The connective tissue cells and odontoblasts are also stained with this method, but not deeply, and the nerve fibres can be very easily traced.

Being desirous of seeing if I could stain these fibres by an aniline dye, I made use of Congo red, which has been employed by Nissl and others for tracing axis cylinders of nerves. I found that a concentrated solution of Congo red in water, employed for one minute, gave appearances exactly like those obtained by the iron haematoxylin process, but the stain was more general and diffused than in the iron process.

A prolonged staining with a weak solution of Congo red (1 in 200 to 1 in 400), as recommended by Nissl (12), gave a somewhat sharper and clearer image, especially when the stain was turned blue by treatment with acid. Some of these sections in which the pulp only had been blued by the acid, the dentine remaining red, were very instructive.

This stain does not appear to be at all permanent in balsam, at all events when treated with the usual clearing reagents, but keeps fairly well in Farrant's solution. To retain the blue color which is the more permanent condition of the stain, the Farrant solution must be rendered distinctly acid.* I find, however, that many of the best stained specimens, mounted in Farrant, have faded badly in a few months.

A concentrated solution of methylene blue (Ehrlich's *intra vitam* stain)used to stain sections, also shows the nerve-fibres entering the tubes very clearly, but quickly fades unless treated by one of the methods employed to fix this stain. The stain is, however, too diffuse to be employed with much advantage for the nerves of the pulp.

The most successful preparations that I have thus far succeeded in obtaining are, however, those prepared with chloride of gold, both ground and decalcified sections.

The ground section, which had been treated by the Weil method and ground down on a stone when impregnated with hardened balsam, was placed in chloroform until all

*Heidenhain believes that the addition of Congo red to albumin solution leads to the formation of a salt in which the albumin plays the part of an acid, while the undissociated color salt takes on the part of a base, there being formed Congo-sodium albuminate. By the addition of acids, the unstable Congo-sodium albuminate is believed to be converted into the stable albumin-Congo sulphonate. The union between albumin and Congo red is so firm that even 5 per cent, sulphuric acid does not always liberate the free blue Congo acid. (Mann, "Physiological Histology," p. 455).

the balsam was thoroughly removed, and then treated with Lowit's formic acid and chloride of gold method. In this specimen the fibres in the tubes, the plexus at the margin, the plexus of Raschkow, and the beaded fibres passing to the dentine are all very clearly shown (figs. 3 and 4).

With the decalcified teeth, I used Ranvier's modification of Lowit's method, staining small pieces of the decalcified teeth in bulk and cutting them on the freezing microtome. I was able to obtain some very thin sections, which showed all the above points very clearly; in the zone of partial calcification in a young tooth the nerve-fibres are deeply stained and seen in remarkable abundance, extending into the dentine and traversing the dentinal tubes.

This being a true differential stain the other elements of the pulp are not colored by it, but by counter-staining with eosin the stained red dentinal fibril can be seen entering the dentinal tubule with the nerve-fibres.

At the suggestion of Professor Schafer, I also treated small pieces of decalcified teeth with the silver nitrate and hydrokinone method of Cajal. In successful preparations the marginal plexus and penetration of the dentine are very well shown.

The conclusions I have arrived at after a careful study of several hundred preparations are as follows:

The medullated nerve-fibres which form the main nerve-trunks of the dental pulp traverse it from their point of entrance at the apical foramen, pursuing a course more or less parallel to the long axis of the tooth, and are closely associated with the blood-vessels. They divide and sub-divide, and the smaller divisions of the medullated fibres, when they approach the periphery of the pulp, lose their medullary sheath, and continue as axis cylinders only, the ultimate nerve-fibres of which these are composed combining in an intricate plexus beneath the odontoblast layer, the plexus of Raschkow.

In good sections cut longitudinally, in which the section has been parallel to the nerves and blood-vessels, several large nerve-trunks can be seen traversing the pulp and, while giving off numerous small side branches in their course, undergoing very little dimunition in size until they approach the periphery of the pulp, immediately beneath the odontoblast layer. In this situation they very suddenly break up into a multitude of fine fibres which are seen to run parallel to the surface of the pulp and give off branches to the plexus of Raschkow.

In this section a nerve-trunk is seen to travel up the centre of the pulp of a bicuspid, exactly between the two cornua; here it divides into several branches which pass out right and left to the cornua; continuing a course parallel to the layer of odontoblast cells and sending off multitudes of fine fibres into the plexus immediately beneath these cells. The much discussed "basal layer of Weil" I believe from an examination of these specimens, to be occupied, as had been previously surmised, by the nerve-fibres forming the plexus, supported by and blended with the delicate connective tissue of the pulp, which passes to the dentine and there becomes incorporated with the dentine matrix as described in my former paper published in the *Philosophical Transactions* for 1891 (14, 15). Sometimes a bundle of medullated fibres in the pulp is seen to spread out in a radiating mass of fine fibrillae, like a brush (see fig. 1.) This appearance has also been figured in Rose and Gysi's (16) "Portfolio of Photomicrographs."

The plexus of Raschkow consists exclusively of non-medullated fibres. Morgenstern described medullated fibres as actually entering the dentine, but the appearances sometimes produced by the method of Golgi probably led to this error.

From the plexus of Raschkow, these fine fibres are seen to pass between and around the odontoblast cells, which are often enclosed in a network of fine nerve-fibres, but they do not appear to make any direct connection with these cells, passing out beyond them to the dentine at the dentine margin; they also run laterally, forming a narrow plexus

**To illustrate article "On the Distribution of the Nerves of the Dental Pulp," by J. Howard Mummery, M.R.C.S., L.D.S.**

From the Phil. Trans. R.S. B. Vol. 202, plate 18.

Fig. 1.

Fig. 2.

Fig. 3.

To illustrate article "On the Distribution of the Nerves of the Dental Pulp," by J. Howard Mummery, M.R.C.S., L.D.S.

Fig. 4

Fig. 5

Fig. 6

Fig. 7

From the Phil. Trans. R.S. B. Vol. 202, plate 18.

in this situation. This narrow plexus is described by Otto Fischer and others as the terminal nerve plexus of the pulp. It was also described by Kolliker, but he said that, although the nerves formed a plexus here, it did not appear to be their real termination. These specimens show that it is not their real termination, but that a multitude of fibres pass out from this plexus into the tubules of the dentine. They may be seen especially well in the gold preparations, entering the dentine in great numbers, and in thin sections there appear to be two or more to each tube.

These beaded fibres can be traced in many preparations to the cemental and enamel margins, where they can be seen as exceedingly fine dots; in the case of the cementum they appear to terminate in these fine arborisations just beneath the granular layer. They are much more difficult to trace to the enamel margin, but in several gold preparations some of the tubes are seen to be filled with fine dotted lines completely to the enamel margin (the enamel having disappeared in the process of decalcification.)

In some few instances, I have seen fine dotted lines upon the separated fibrils projecting from the pulp, but have never been able to see the beaded fibres projecting from the surface unless supported in this way.

### SUMMARY.

The points I have endeavored to prove in this communication are as follows:—

(1) That the fine nero-fibrils of the pulp, after interlacing in a plexus beneath the odontoblasts (the plexus of Raschkow), pass between and around the odontoblasts cells and form a narrow plexus at the inner margin of the dentine, which might be termed the *marginal* plexus.

(2) That from this marginal plexus the nerve-fibres pass into the dentinal tubules, which they traverse in company with the dentinal fibril.

(3) That these fibrils end in arborisation beneath the enamel and cementum, following the fine terminal branches of the dentinal tubules.

As these minute neuro-fibrils pass along the tubules of the dentine in their final ramifications, and these tubules are seen in many cases to cross the dentine-enamel margin and end in the enamel, it is possible that many fine nerve fibres pass a short way into the enamel with them, but I have not been able to stain any nerve-fibres in the calcified enamel.

This would not appear to be any syste natic innervation of the ena nel, as appears to be suggested by Morgenstern and Romer, but more in the nature of an accidental penetration of the tissue by nerve-fibres. If the nerve-fibres terminate in the so-called enamel spindles, it is very difficult to understand why these spindles are not more regularly distributed, and why they seem to be entirely absent in the enamel in some teeth. There is no doubt whatever, whether they are spaces filled with air, as some think, or filled with protoplasmic material, that the dentinal tubules can often be traced into them.

The teeth being dermal structures and the enamel being an epithelial tissue, the mode of distribution of the nerves of the dental pulp, as described in the present communication, would appear to be quite in harmony with the usual arrangement of the nerve-fibres in their distribution to other epithelial tissues.

Professor Schafer (17) in his "Essentials of Histology," says: "When sensory nerve-fibres terminate in epithelium, they generally branch once or twice in the sub-epithelial connective tissue on nearing their termination. The sheaths of the fibres then successively become lost, first the connective tissue or perineural sheath, then the medullary sheath, and lastly the neurolemma, the axis cylinder being alone continued as a bundle of primitive fibrils. This branches, and with the ramifications of the axis cylinders of the neighboring nerve-fibres forms a primary plexus.

"From the primary plexus smaller branches come off, and these form a secondary plexus nearer the surface, generally immediately under the epithelium if the ending is in a membrane covered by that tissue. Finally, from the secondary plexus nerve-fibres proceed and form terminal ramifications among the tissue-cells, the actual ending being in free varicose fibrils. This mode of ending is characteristically seen in the cornea of the eye, but can also be rendered evident in other epithelia."

### METHODS OF PREPARATION.

For the fixation of the nerve-tissue of the pulp, I find formalin preferable to all other fixing agents. It is best used in a 4 per cent. solution of formaldehyde, that is, 10 parts of the 40 per cent. commercial formalin solution to 90 parts of water. (Teeth that have been cut across just below the neck should be kept in this solution about a week, but a longer period is not harmful.)

To decalcify the teeth the most useful fluid is 3 per cent. nitric acid; this will usually soften a bicuspid tooth in about three days. The tooth should be placed in at least 100 c.c. of the solution, which should be changed every twenty-four hours. When sufficiently softened the teeth should be treated for half an hour with a solution of carbonate of lithia or carbonate of soda (5 grains to 1 oz.), and washed thoroughly in distilled water.*

They are then left for twenty-four hours, or until they sink to the bottom of the solution, in a strong solution of dextrin (which I find preferable to gum arabic), and cut on the freezing microtome. The knife should be kept very sharp, and any very thin and small sections carefully preserved, as, especially with gold and silver nitrate preparations, the very thin edges of dentine obtained in this manner are very valuable for the demonstration of nerve-fibres. They may also be embedded in paraffin, but thinner sections can usually be obtained by the freezing method.

### (*1*) *Iron and Tannin.*

Sections prepared as above are placed in:—

(1) Solution of perchloride of iron in water, 4 per cent.

(2) Well washed and transferred to—

(3) Solution of tannic acid in water, 2 to 4 per cent., and carefully watched until they are sufficiently blackened.

(4) Well washed, dehydrated, cleared, and mounted in balsam (pyrogallic acid may be used instead of tannic acid).

---

*I have lately abandoned the use of nitric acid as a decalcifying agent and have employed formic acid, 33·3 per cent., which rapidly decalcifies the teeth and does not cause the shrinking of the odontoblast cells so noticeable with nitric acid.

*(2) Iron Haematoxylin (Benda).*

Sections cut on the freezing microtome are—

(1) Placed first in the mordanting solution, which is composed as follows:—

| | |
|---|---|
| Sulphate of iron | 80 parts. |
| Water | 40 " |
| Sulphuric acid | 15 " |
| Nitric acid | 18 " |

This solution, which contains 10 per cent. of iron, should be diluted when used with one or two volumes of water. Sections are kept in the mordanting solution for twenty-four hours.

(2) Wash first in distilled, then in tap water.

(3) Place in a 1 per cent. solution of haematoxylin in water until quite black.

(4) Differentiate, by placing in a 10 per cent. solution of acetic acid in water, examining from time to time to see if sufficiently cleared.

The sections may be mounted in balsam or in Farrant solution (*a*).

*(3) Congo Red.*

(1) Sections may be stained for one minute in a concentrated solution of Congo red in water.

(2) Washed with distilled water.

(3) Treated for a few minutes with 5 per cent. hydrochloric acid in water until a good blue color.

(4) Mount in acidulated Farrant's solution.

Or sections may be stained in a weak solution of Congo red, five parts in 400 of water, and left in the staining fluid for forty-eight hours.

If mounted in balsam, the sections should be passed through alcohol, and then placed for a sufficient time in 3 per cent. nitric acid in alcohol, to turn them a good blue. (*b*).

*(4) Ranvier's Modification of Lowit's Gold Chloride Process.*

Small pieces of tissue, not more than 4 mm. thick, are placed in a mixture of chloride of gold and formic acid (four parts of 1 per cent. gold chloride to one part formic acid), which has been boiled and allowed to cool. The tissue is kept in this solution for four hours or more, and reduced in formic acid (one part acid to four parts water) in the dark. ("By boiling in the presence of the acid, the gold acquires a great tendency to reduction, and for this reason its selective action on nervous tissues is enhanced."—Ranvier [13]).

Sections may be cut on the freezing microtome. (*c*)

*Cajal's Method.*

(1) Small pieces of the decalcified tooth, not more than 4 mm. thick, are placed in 50 c.c. of rectified spirit, to which three or four drops of ammonia may be added, and kept in this solution for from four to six hours.

(2) Transferred to absolute alcohol for twenty-four hours.

(3) Rinse with distilled water.

---

(*a*) See Lee, "Microtomist's Vade Mecum," p. 176, ed. 1896.

(*b*) Nissl, *loc. cit.* (*c*) B. Lee, *loc. cit.*

(4) Place in a large quantity of 1.5 per cent. solution of silver nitrate, and keep in warm incubator at about 35°C. for five or six days.

(5) Rinse in distilled water for a few seconds.

(6) Place in the following solution for twenty-fours hours:—

| | |
|---|---|
| Hydrokinone | 1—1.5 grm. |
| Distilled water | 100 c.c. |
| Formol | 5—10 c.c. |
| Rectified spirit | 10—15 c.c. |

(7) Wash in water for some minutes.

(8) Cut sections and mount. (*d*)

(Very small pieces should be taken, as I find it very difficult by this process to get through penetration of the dentine; the first few sections cut from the small pieces of tissue employed are well impregnated, but deeper in there is no impregnation, although a much longer time was given in both solutions than stated above.)

### LITERATURE

(1) "Bau und Entwickelung der Mundhoehle des Menschen," Leipzig, 1909, p. 311.

(2) "Essentials of Histology," 1910 edition, p. 3106.

(3) "Morphologie du Follicule dentaire chez les Mammiferes," *Journal de l'Anatomie et Physiologie*, 1879.

(4) A. Hopewell-Smith. *Odontological Transactions*, 1893, and "Histology and Patho-Histology of the Teeth," 1903, p. 170, and Appendix.

(5) "Untersuchungen ueber die Zahnpulpa," *Archives f. Microskop. Anat.*, vol. iv., p. 73.

(6) Retzius. "Biologische Untersuchungen," *Neue Folge*, vol. vi., p. 64.

(7) "The Innervation of Tooth Pulp," *Dental Cosmos*, 1898, vol. xl., p. 803.

(8) Morgenstern. "Ueber das Vorkommen von Nerven in den harten Zahnsubstanzen," *Deutsche Monatschrift f. Zahnheilkunde*, 1892, p. 436, and ditto, 1895, p. 111.

(9) Oscar Romer. "Zahnhistologische Studie," 1899 (quoted by Hopewell-Smith, *loc. cit.*, p. 164.)

(10) C. S. Tomes. "A Manual of Dental Anatomy," 6th edition, 1904, pp. 63 and 98.

(11) *Proceedings of the Royal Society of Medicine*, April, 1911.

(12) Nissl. *Zeit. f. Wiss. Mik.*, III., 1886, vol. iii., p. 398.

(13) Bolles Lee. "Microtomist's Vade Mecum," 1896, edition, p. 293.

(14) Von Ebner. *Handbuch der Zahnheilkunde*, Wien, 1890-91.

(15) J. H. Mummery. "Some Points in the Structure and Development of Dentine," *Philosophical Transactions*, 1891, vol. clxxxii., pp. 527-45.

(16) C. Rose and Gysi. "Portfolio of Microphotographs of Dental Histology," 1895.

(17) Schafer. *Loc. cit.*, p. 199.

### DESCRIPTION OF PLATES.

Fig. 1.—A bundle of medullated fibres accompanying a blood-vessel at the coronal portion of the periphery of the pulp. The axis cylinders

(*d*) Shaefer, *loc. cit.*, p. 561.

are seen breaking up into a multitude of fine neuro-fibrils which are given off to the plexus beneath the odontoblast layer. From a ground transverse section of a human bicuspid tooth prepared by the Weil process and stained with iron and tanning. X180.

Fig. 2.—From a decalcified preparation of a human bicuspid tooth stained with iron haematoxylin (Benda), transverse section. (*d*) Dentine; (*c*) the partially calcified area; (*n*) fine fibrils from the marginal plexus passing into the dentinal tubules; (*p*) pulp and nerve plexus. X650.

Fig. 3.—From a ground transverse section of a human bicuspid tooth prepared by the Weil process and stained with gold chloride (Lowit). (*d*) Fully calcified dentine, showing the fine beaded fibrils in the tubules; (*c*) partially calcified area crossed by fine neuro-fibrils; (*p*) pulp and marginal plexus. X850.

Fig. 4.—From another part of the same preparation as fig. 3. (*d*) Fully calcified dentine; (*c*) partly calcified area; (*m*) marginal plexus; (*p*) pulp and nuclei of odontoblasts. The beaded fibres are seen in great numbers entering the marginal plexus (*m*) and passing along the tubules in the partially calcified area (*c*) to the hard dentine (*d*). In these ground preparations there is no shrinkage of the elements of the pulp apparent, the nuclei retaining their form. X850.

Fig. 5.—From the transverse section of a decalcified human bicuspid tooth taken from a very thin margin of the section—treated with nitrate of silver and pyridin for four days at a temperature of 40° C., and reduced with pyrogallic acid. The odontoblasts are considerably shrunk, but the nerve-fibres are clearly seen passing from the plexus of Raschkow (*n*) to the marginal plexus (*m*) and from this plexus entering the dentinal tubules as distinct beaded fibres. (*d*) Dentine; (*c*) partially calcified area; (*m*) marginal plexus; (*n*) plexus of Raschkow. X800.

Fig. 6.—From a decalcified section of a human bicuspid tooth, showing the two rows of beaded fibres in the tubules in the substance of the dentine, stained with gold chloride (Ranvier). X650.

Fig. 7.—From a longitudinal section of an unerupted human bicuspid tooth treated by Ramon y Cajal's silver nitrate method. (*d*) Fully formed dentine; (*c*) a very wide partially calcified area; (*n*) beaded fibres stained black with the silver nitrate entering the tubes; (*p*) pulp. The connective tissue and other elements of the pulp stained yellow-brown. X1,200.

—*Dental Record.*

## Figures and Tables

**Fig. 1. f1:**
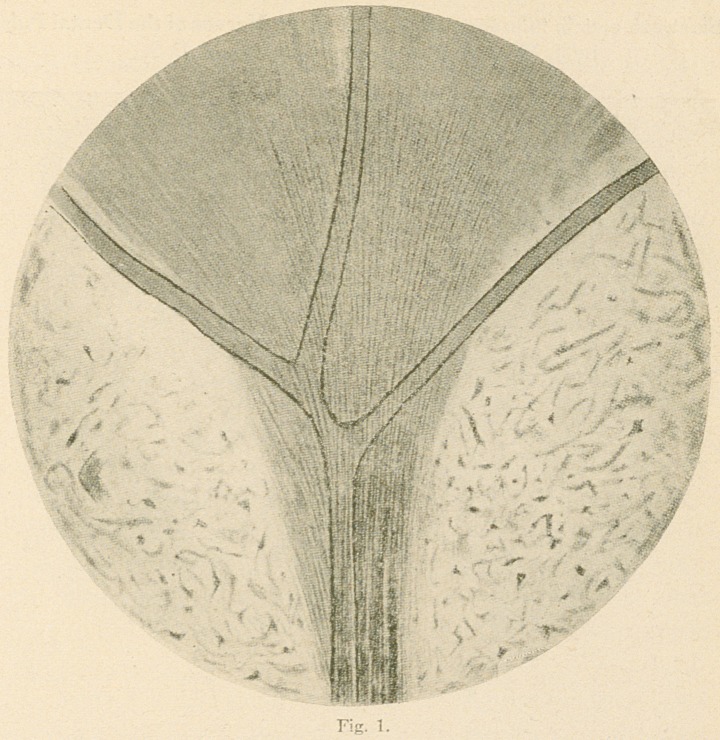


**Fig. 2. f2:**
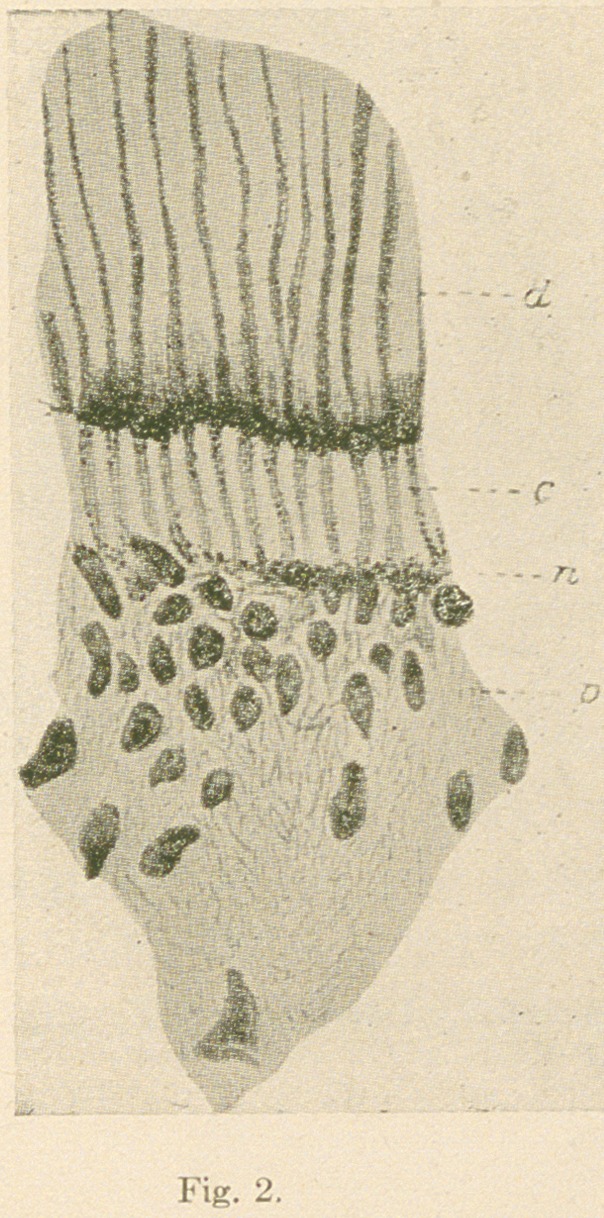


**Fig. 3. f3:**
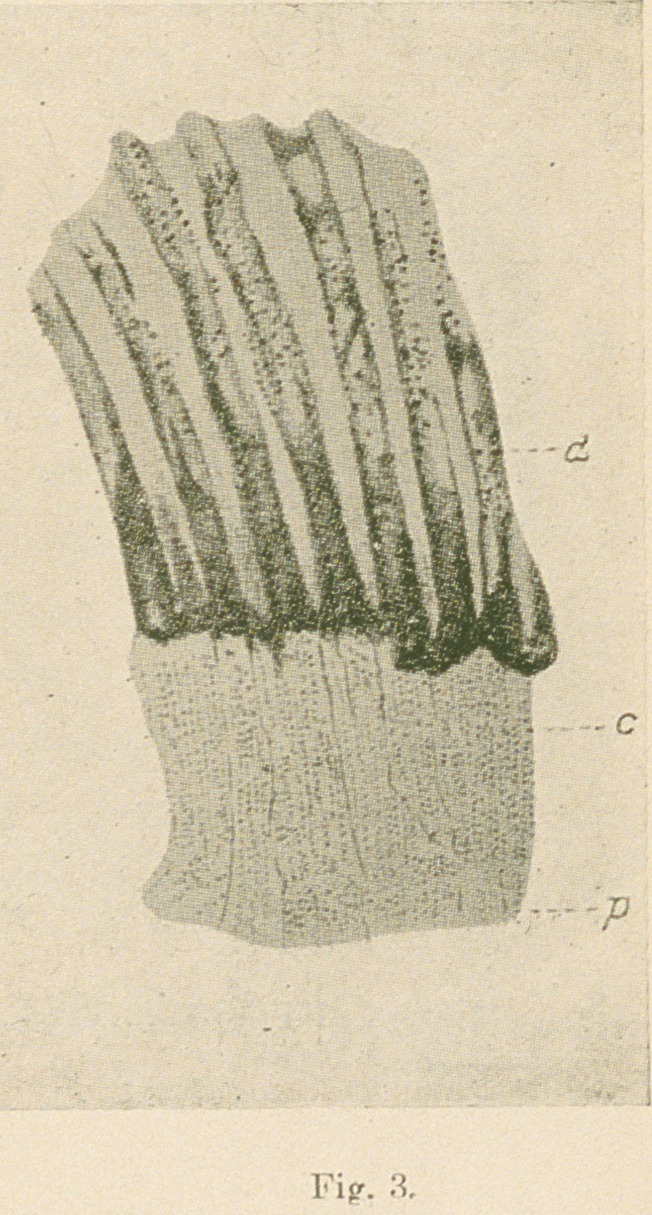


**Fig. 4. f4:**
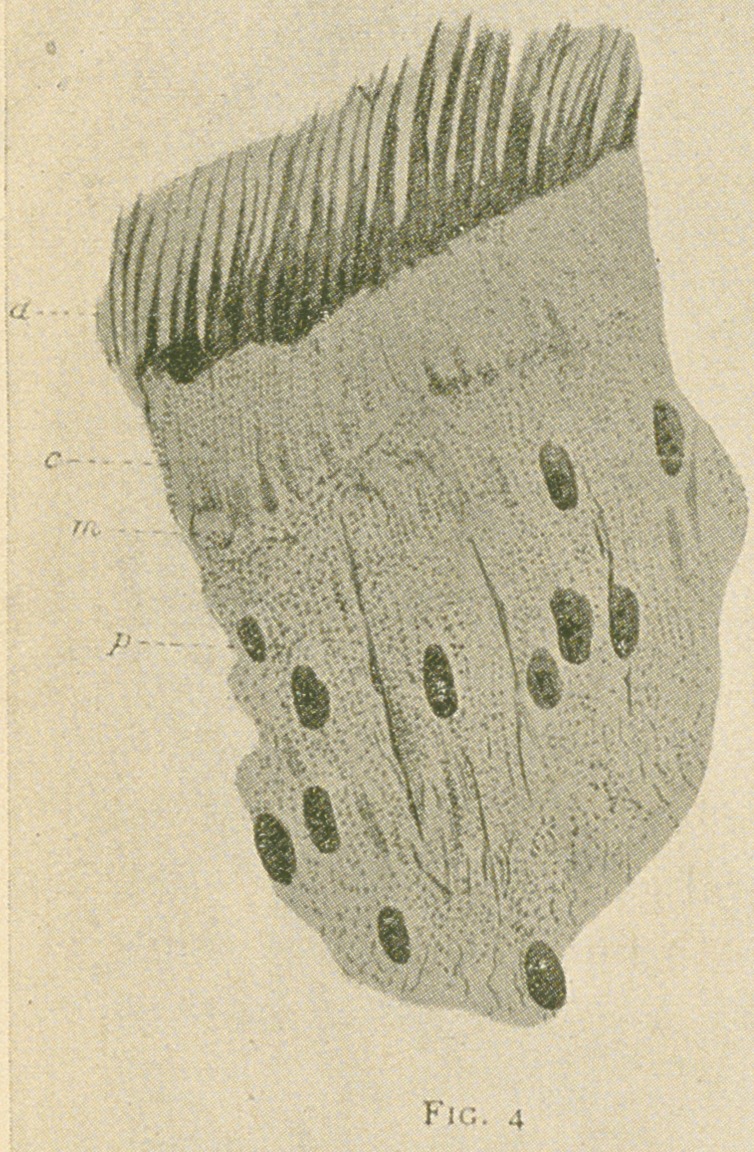


**Fig. 5. f5:**
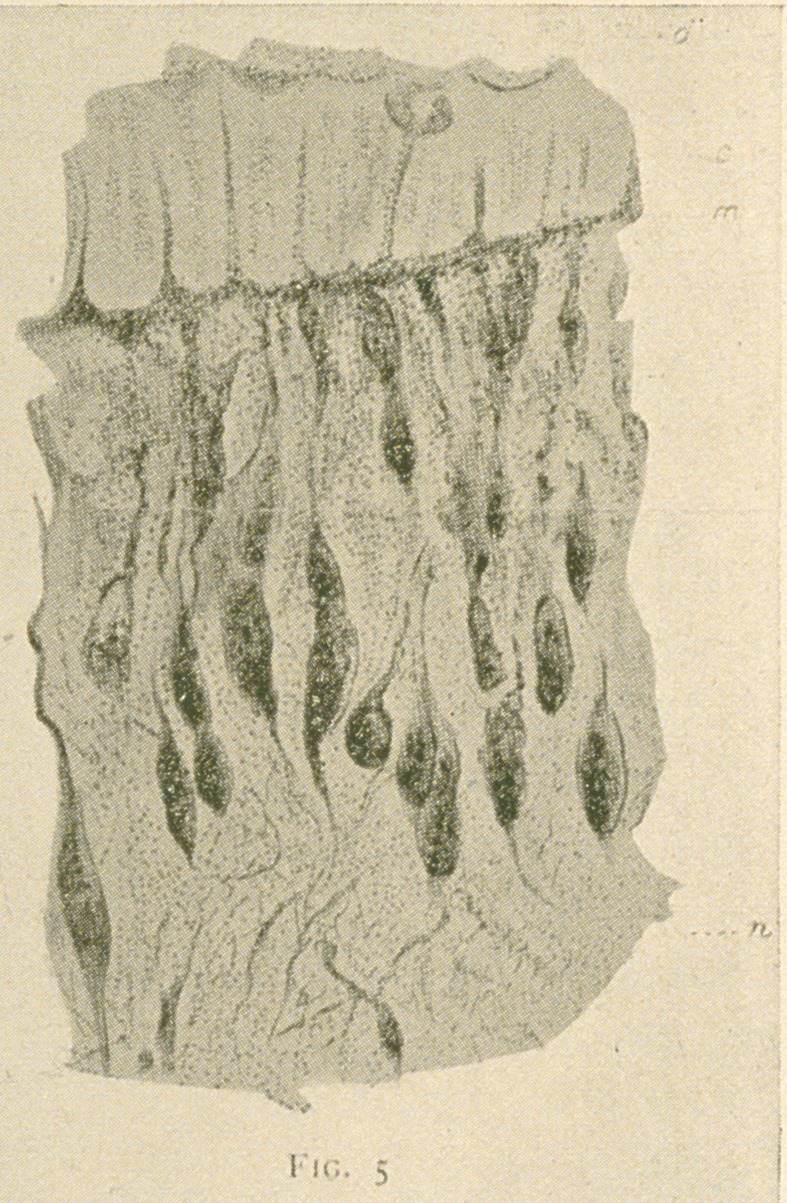


**Fig. 6. f6:**
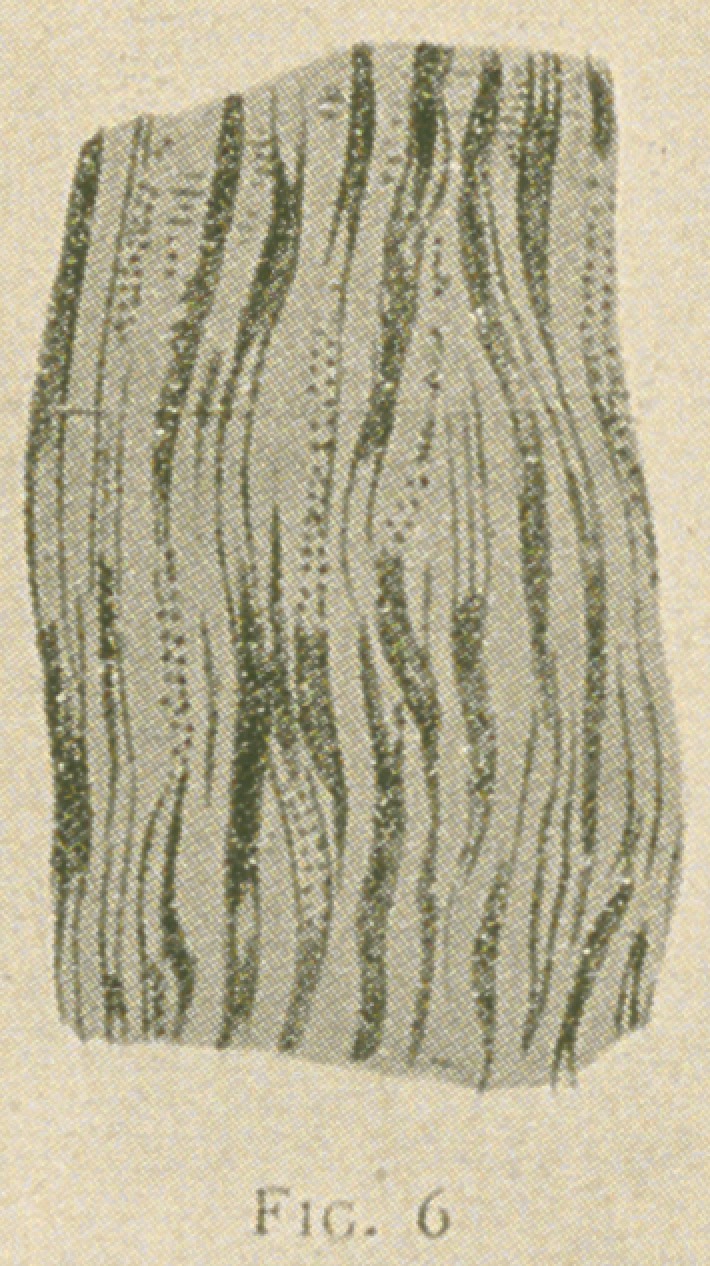


**Fig. 7. f7:**